# Screening of Chilli Pepper Genotypes as a Source of Capsaicinoids and Antioxidants under Conditions of Simulated Drought Stress

**DOI:** 10.3390/plants9030364

**Published:** 2020-03-16

**Authors:** Tomas Kopta, Agnieszka Sekara, Robert Pokluda, Vojtech Ferby, Gianluca Caruso

**Affiliations:** 1 Department of Vegetable Growing and Floriculture, Faculty of Horticulture, Mendel University, 613 00 Brno, Czech Republic; robert.pokluda@mendelu.cz (R.P.); xferby1@node.mendelu.cz (V.F.); 2 Department of Horticulture, Faculty of Biotechnology and Horticulture, University of Agriculture, 31-120 Krakow, Poland; agnieszka.sekara@urk.edu.pl; 3 Department of Agricultural Sciences, University of Naples Federico II, 80055 Portici (Naples), Italy; gcaruso@unina.it

**Keywords:** *Capsicum*, cultivar, stress, soluble carbohydrates, ascorbic acid, phenolics, radical scavenging activity, Scoville Heat Units

## Abstract

In many regions of the world, the production of vegetable crops is limited by a deepening water crisis. Drought stress affects productivity and the chemical composition of crops. The variability of drought tolerance between species and cultivars of economically important crops, such as pepper (*Capsicum* species), requires specific investigations to understand the physiological and biochemical responses to the aftermath of drought. The fruits and leaves of four chilli pepper cultivars were investigated to elucidate the fruits’ pungency (Scoville Heat Units, SHU), ascorbic acid content, DPPH (2,2-diphenyl-1-picrylhydrazyl) radical scavenging activity, polyphenol content, membrane lipid peroxidation and key protective antioxidant enzyme activity under drought stress (18–28% volumetric water content) as compared to the control (35–60%). Drought increased the chilli pepper fruits’ pungency expressed in Scoville Heat Units (SHU) as well as ascorbic acid content, but this relationship was also dependent on genotype and stress duration. ‘Jolokia’ was marked as most sensitive to drought by increasing content of capsaicinoids and DPPH˙ scavenging activity under stress conditions. Capsaicinoids and Ascorbic acid (AsA) greatly influenced the antioxidant activity of highly pungent chilli pepper fruits, although total phenols played a significant role in the mildly pungent genotypes. Generally, the activities of antioxidant enzymes increased under drought in chilli pepper leaves and fruits, although the intensity of the reaction varied among the cultivars used in the current research. All the investigated biochemical parameters were involved in the drought response of chilli pepper plants, but their significance and effectiveness were highly cultivar-dependent.

## 1. Introduction

Sweet and hot peppers are among the most important vegetables and spices in the human diet, also used in the pharmaceutical and pesticide industries due to their high content of capsaicinoids, unique alkaloids restricted to the *Capsicum* species. Peppers were domesticated in the Americas and successively introduced into cultivation in almost all the regions of the world [1]. In the Central European climatic zone, peppers are grown under protected conditions, in greenhouses or foil tunnels, to produce high yields of the best quality fruits, intensively coloured and rich in taste-strengthening compounds. Pepper is commonly planted in the soil or in containers filled with soil- or peat-based substrates, fertigated, and bell pepper canopy training is a common practice. The chilli pepper is cultivated without training. The container growing cost is higher, but it decreases the risk of adjacent plant contamination with root-borne diseases [2,3].

Chilli pepper is added to dishes in small amounts, but the intake of chilli even as a spice enriches the diet with polyphenols and other bioactive compounds, especially ascorbic acid and other vitamins, carotenoids (α- and β-carotene), capsaicinoids and mineral salts [4]. The most characteristic phytochemicals of all members of the genus *Capsicum* are capsaicinoids, compounds providing the pungent taste but also of exhibiting antioxidant activity [5]. There is a growing interest in the enhancement of compounds in food having health-promoting attributes such as antioxidants, and which were previously regarded as non-nutritive, including phenolic compounds (simple phenols, flavonoids, anthocyanins, lignans and lignins, stilbenes and tannins). Phenols are antioxidants in nature, often associated with plant defence against biotic and abiotic stress factors. Additionally, phenols modify fruit colour, taste, aroma and flavour, and also provide health-beneficial effects for humans [6,7].

Variability in the presence and concentration of phytochemicals in pepper species can be a factor affecting the selection of genotypes for breeding programmes [8]. Fruit chemical composition can also be managed by targeted stress application and growing practices [9]. Pepper is sensitive to drought stress. In the regions with limited water resources, the rate of applied water can be decreased only by around 20% without significant yield loss [10]. Generally, drought stress can induce cellular damage due to the accumulation of reactive oxygen species (ROS), leading to disturbance of redox homeostasis in plant cells and the production of malondialdehyde, the final result of lipid peroxidation in the cellular membranes, which is taken as an index of oxidative membrane damage [11]. Among the major ROS-scavenging enzymes, catalase (CAT), superoxide dismutase (SOD) and ascorbate peroxidase (APX) are mentioned, acting in chloroplasts, mitochondria, peroxisomes and cytosol to protect cell compartments against oxidative stress [12]. However, APX may have a pivotal role in ROS-scavenging because even very low concentrations of APX are sufficient for H_2_O_2_ decomposition. Ascorbic acid, an important compound in pepper fruits, acts as a primary substrate for enzymatic detoxification of hydrogen peroxide [13]. A prominent response to drought stress is the accumulation of substances counteracting the loss of turgor and modulating the osmotic potential of the cell’s cytosol and the vacuoles, including amino acids, polyamines, glycine betaine, hydrophilic proteins, carbohydrates and polyols [14,15]. Many stress-induced metabolites act as antioxidants, increasing the plant’s environmental adaptation as well as the nutritional and pharmacological value of crops in the human diet [16].

Drought is recognised as a stress factor that increases capsaicinoid accumulation in pepper fruits, although low pungency cultivars seem to be more sensitive in this respect [17], although Jeeatid et al. [18] determined that cultivars with larger fruit size and higher pungency produced more capsaicinoids in water stress conditions. Concerning the stage of development, *Capsicum* spp. are more susceptible to the aftermath of drought at the vegetative stage than at either the fruiting or the flowering stages [19]. Proper water management seems to be extremely important at all stages of pepper development to avoid physiological disturbances, disease problems and decreases in fruit set and quality, although there are very limited data on mechanisms of drought adaptation in chilli pepper cultivars. Additionally, the chilli pepper’s physiological and biochemical response to drought intensity and duration is a good criterion for evaluating cultivars with high capsaicinoid content [20].

Since the pepper is considered one of the most sensitive crops to soil water deficit, we hypothesised that application of moderate drought stress to greenhouse-grown, potted plants of different cultivars would allow the analysis of the mechanisms of biochemical and physiological adaptation. The response of leaves and fruits were included to track the pathways of adaptation linked with the fruit’s biochemical profile, the most important component of its quality. 

## 2. Results

### 2.1. Scoville Heat Units (SHU) Values and Ascorbic Acid (AsA) Content in Chilli Pepper Fruits

Results from analysis of variance (ANOVA) (Table 1) clearly show that SHU and AsA content were significantly affected by all the three experimental factors and by their interactions, except for the non-significant interaction between drought stress and its duration on SHU values.

**Figure 1:**
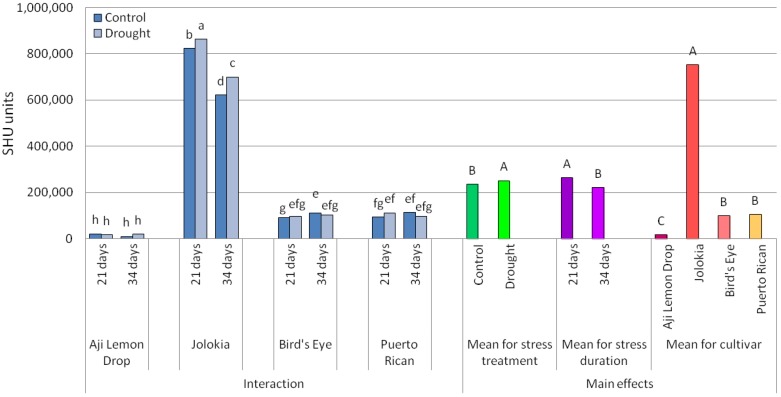
SHU values of chilli pepper fruits depending on cultivar, drought stress and drought duration. The data gives the mean values of three replications. Different letters denote significant differences: lowercase letters refer to the interaction and capital letters to the main effects (Scheffe’s test, *p* ≤ 0.05).

**Figure 2:**
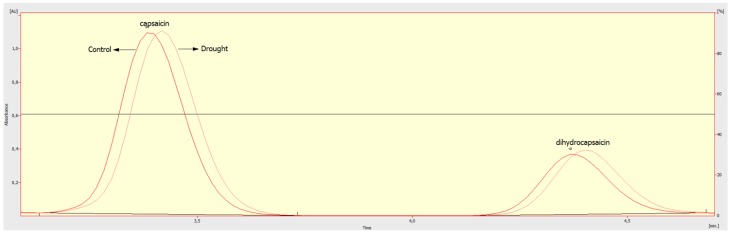
Chromatogram of the capsaicin and dihydrocapsaicin contents in the Jolokia cultivar under control and drought stress.

The SHU values showed ‘Jolokia’ fruits having the highest pungency and ‘Aji Lemon drop’ fruits having the lowest (Figure 1 and Figure 2). SHU values were determined at a comparable level on fruits of ‘Bird´s Eye’ and ‘Puerto Rican’. The main effects of drought stress application and duration were significant (Table 1). Under drought conditions, a higher average level of SHU was determined. Regarding drought stress duration, it was shown that a 21-day-long water deficit caused higher SHU values compared to 34-day-long drought application. 

Interactions between all experimental treatments on fruit pungency were also evaluated. The ‘Bird´s Eye’ and ‘Puerto Rican’ fruits showed a tendency to have higher SHU levels under drought stress conditions applied for 21 days, while 34-day-long stress treatment caused the reverse effect. ‘Jolokia’ fruits were characterised by significantly higher SHU levels under drought conditions regardless of duration. Differences between the two stress durations in the ‘Aji Lemon Drop’ were not significant. 

**Figure 3:**
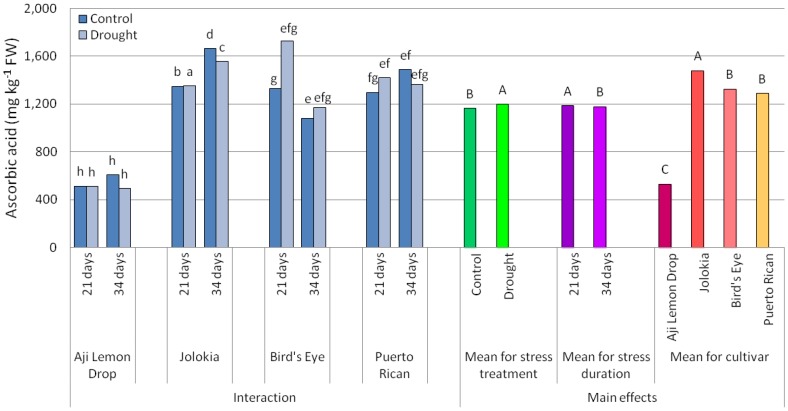
Ascorbic acid content (mg kg^−1^ fresh weight (FW)) in chilli pepper fruits depending on cultivar, drought stress and drought duration. Data are the mean values of three replications. Different letters denote significant differences: lowercase letters refer to the interaction and capital letters to the main effects (Scheffe’s test, *p* ≤ 0.05).

The lowest amount of ascorbic acid was detected in ‘Aji Lemon Drop’ fruits (Figure 3). The remaining cultivars reached values around 1000–1600 mg per 1000 g^−1^ of fresh weight (FW). The analyses of the main effects (Table 1) showed that generally, drought stress increased AsA content in chilli pepper fruits. Drought application for 21 days resulted in a higher content of AsA compared to 34-day-long stress treatment.

The interaction between cultivar, drought stress application and duration on ascorbic acid content were also significant. After a 21-day-long period of drought, AsA content was higher in the ‘Bird´s Eye’ and ‘Puerto Rican’ fruits compared to the amount determined after 34 days of stress treatment. ‘Aji Lemon Drop’ and ‘Jolokia’ showed no statistical difference in AsA content. ‘Bird´s Eye’ fruits, sampled from plants treated with drought for 21 days, contained a 130% higher amount of AsA compared to those exposed to water deficit for 34 days. The only cultivar with a significantly higher AsA level after 34-day-long drought treatment was ‘Bird´s Eye’. The remaining cultivars showed a significant decrease in AsA in fruits of stressed plants compared to those grown in optimum conditions.

### 2.2. Stress Parameters of Chilli Pepper Fruits and Leaves

Fruits of chilli pepper cultivars were not differentiated concerning total phenols (TP) content in drought stress and control conditions (Table 2). The different effect of water deficit was recorded for TP in leaves, namely, drought conditions caused a significant increase of these compounds in ‘Aji Lemon Drop’, ‘Jolokia’ and ‘Bird’s Eye’ leaves, and a decrease in ‘Puerto Rican’. Generally, a higher TP content was determined in the leaves and fruits of ‘Jolokia’ while the lowest was in ‘Aji Lemon Drop’. Drought stress caused a decrease in soluble carbohydrates (SC) content in ‘Aji Lemon Drop’ fruits but for ‘Jolokia’, the reverse effect was noted. The ‘Bird’s Eye’ and ‘Puerto Rican’ fruits contained similar levels of SC in control and stress conditions. Analysis of the main effects showed that ‘Jolokia’ fruits contained the highest amount of SC, followed by ‘Puerto Rican’, ‘Bird’s Eye’ and ‘Aji Lemon Drop’. SC content was not statistically differentiated in the leaves of all chilli pepper cultivars.

‘Jolokia’, ‘Bird’s Eye’ and ‘Puerto Rican’ fruits sampled from drought-stressed plants were characterised by higher DPPH˙ scavenging activity as compared to those collected from control plants. ‘Bird’s Eye’ and ‘Puerto Rican’ leaves collected from drought-stressed plants showed higher DPPH˙ scavenging activity in comparison to the control ones. Generally, higher DPPH˙ scavenging activity was noted in ‘Jolokia’ fruits and leaves while the lowest was in ‘Aji Lemon Drop’ fruits and leaves.

Antioxidant enzymes activity and malondialdehyde content were significantly differentiated between cultivars and stress treatment in chilli pepper fruits and leaves as well (Table 3). Catalase (CAT) activity analysed in ‘Aji Lemon Drop’ fruits was highest in fruits collected from plants exposed to drought stress as compared to control ones. No significant differences were recorded in the fruits from the other three cultivars, showing about a 7 times lower CAT activity than ‘Aji Lemon Drop’. Significantly higher CAT activity was observed in leaves of drought-stressed plants of all cultivars as compared to the controls. However, except for ‘Aji Lemon Drop’, the values of leaf CAT activity were much higher than those detected in fruits and the cultivars ranking referred to the two plant parts are very different from each other.

Ascorbate peroxidase (APX) activity determined in ‘Aji Lemon Drop’, ‘Bird’s Eye’ and ‘Puerto Rican’ was higher in drought-treated fruits compared to the controls. A similar relationship was observed in the leaves of all cultivars. Generally, ‘Aji Lemon Drop’ was characterised by a higher APX activity measured in fruits and leaves.

‘Jolokia’, ‘Bird’s Eye’ and ‘Puerto Rican’ fruits sampled from drought-treated plants showed higher guaiacol peroxidase (GPX) activity as compared to the controls. Similar results were determined in ‘Aji Lemon Drop’, ‘Bird’s Eye’ and ‘Puerto Rican’ leaves. The main effects showed the highest GPX activity in the fruits of ‘Aji Lemon Drop’ and the leaves of ‘Bird’s Eye’ peppers.

Malondialdehyde (MDA) content was significantly higher in the fruits and leaves of all cultivars subjected to drought stress. In general, the highest content of this compound was determined in the fruits and leaves of ‘Aji Lemon Drop’.

### 2.3. Analysis of Correlation Coefficients, Principal Component Analysis and Hierarchical Cluster Analysis of Chilli Pepper Fruit and Leaf Parameters

**Figure 4:**
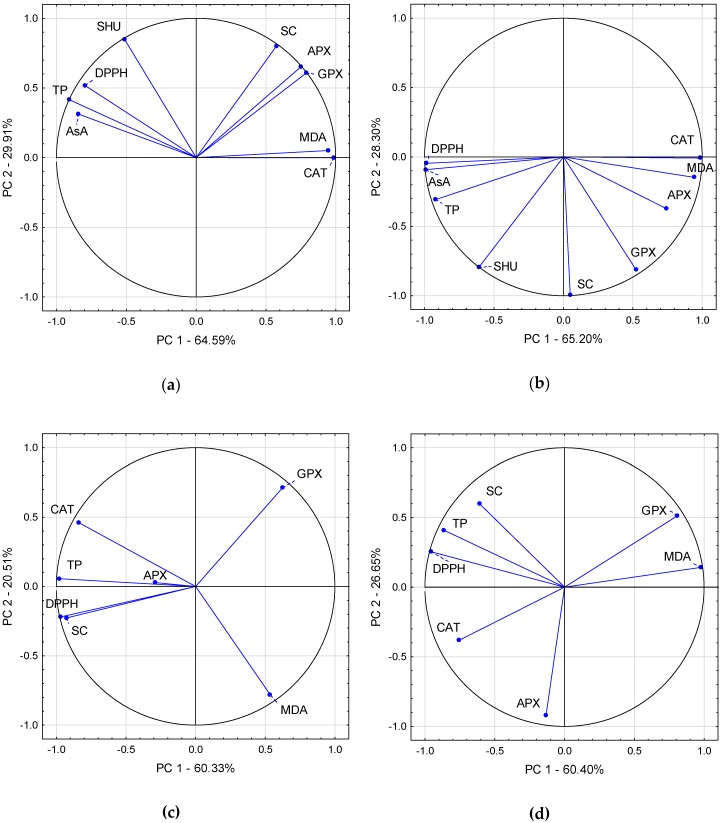
Vector view treatment by trait biplot of principal component analysis outputs showing the interrelationship among measured traits in fruits of control plants (**a**), drought-stressed plants (**b**), and leaves of control plants (**c**) and drought-stressed plants (**d**). SHU—Scoville Heat Units; AsA—ascorbic acid; TP—total phenols; SC—soluble carbohydrates; DPPH—% scavenging of DPPH˙; APX—ascorbate peroxidase; GPX—guaiacol peroxidase; CAT—catalase; MDA—malondialdehyde.

The relationship between experimental traits was determined by Pearson’s correlations analysis (Table 4 and Table 5) and illustrated with biplot graphs based on principal component analysis. Pearson’s correlations showed that SHU levels were positively correlated with AsA, TP and SC content, grouped together in the biplot graph (Figure 4A, B). DPPH-radical scavenging activity was positively associated with SHU, AsA and TP. These relationships were confirmed by the acute angles between their eigenvectors. Antioxidant enzymes, namely APX, GPX and CAT activities, were positively associated with each other and negatively with AsA. All variables were located away from the origin, so they are well represented on the factor map. The presented biplots explained 94.5% of the total variation of the standardised data of chilli pepper fruit parameters for control treatment and 93.5% for drought stress treatment.

DPPH-radical scavenging activity of chilli pepper leaves was positively correlated with TP and SC contents as well as CAT activity, but negatively with GPX activity (Table 5). Biplots explained 80.8% of the total variation of the standardised data of chilli pepper fruit parameters for control treatment and 87.0% for drought stress treatment, so the relationship between the experimental treatments and analysed parameters was more complex and unpredictable for chilli pepper leaves than fruits.

**Figure 5:**
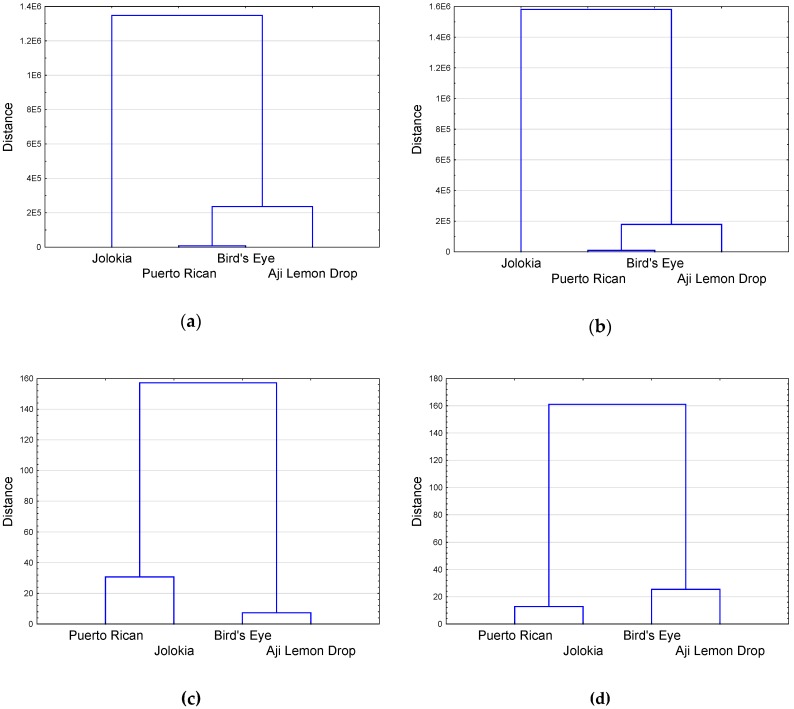
Dendrogram (Euclidean distance, Ward’s method) showing the extent of distances between investigated chilli pepper cultivars based on biochemical similarity matrices from data on fruits of control plants (**a**), drought-stressed plants (**b**), and leaves of control plants (**c**) and drought-stressed plants (**d**).

In the dendrograms in Figure 5a,b, there is a clear distinction between ‘Jolokia’ and the remaining cultivars regarding the chemical composition of fruits. ‘Bird’s Eye’ and ‘Puerto Rican’ appear closely related to each other. Figure 5c,d showed that cultivars can be categorised into two major clusters, closely distanced ‘Bird’s Eye’ and ‘Aji Lemon Drop’, and more distanced ‘Puerto Rican’ and ‘Jolokia’.

## 3. Discussion

### 3.1. Scoville Heat Units (SHU) Values and Ascorbic Acid (AsA) Content in Chilli Pepper Fruits

Chilli pepper cultivars varied to a high degree in values of Scoville Heat Units (SHU), describing their pungency in terms related to the concentration of capsaicinoids, among which, capsaicin is the predominant component [21]. There are five levels of pungency classified using SHU: non-pungent (0–700 SHU), mildly pungent (700–3000 SHU), moderately pungent (3000–25,000 SHU), highly pungent (25,000–70,000 SHU) and very highly pungent (>80,000 SHU). In the present study, the highest SHU values were determined for ‘Jolokia’, which can be classified as highly pungent and the lowest for ‘Aji Lemon Drop’—mildly pungent. Dewitt and Bosland [22] published the SHU values for the ‘Aji Lemon Drop’ of between 30,000 and 50,000 and the cited range is similar to our results. ‘Jolokia’ is known as a very pungent cultivar, showing the SHU range to be about 391,000–1,000,000 [23,24], and our results are similar to the cited values. The SHU value for the ‘Bird´s Eye’ cultivar was reported in the range 100,000–500,000 SHU [25], in line with our results. According to the growers’ website [26], the pungency of ‘Puerto Rican’ is around 250,000–700,000 SHU, which is a higher value than that achieved in our experiment. The results of the present research showed that under drought conditions, the average level of SHU increased. Drought application for 21 days was more effective in capsaicinoid synthesis promotion than longer stress duration (34 days). Among the investigated cultivars, ‘Jolokia’ reacted most intensively to water deficit with increased capsaicinoid synthesis. ‘Jolokia’ can be marked as the most sensitive to drought in terms of increasing pungency under stress conditions.

The mechanisms of crop plants’ reaction to different abiotic stresses are common. Abiotic stresses cause the accelerated production of reactive oxygen species (ROS). Plants have developed common antioxidant defence systems to control the oxidation cascades and to alleviate the cell damage caused by ROS [27]. Non-enzymatic (i.e., capsaicinoids) and enzymatic antioxidants may play an important role in complex mechanisms involving both avoiding ROS overproduction and scavenging of ROS produced. The results from the present research confirmed that drought stress affected capsaicinoid accumulation. Almost all the cultivars showed higher SHU values under drought stress; however, only in the case of ‘Jolokia’ were the differences statistically significant. This finding shows a relationship that is the opposite of that described by Phimchan et al. [17], who stated that the capsaicin content of low- and medium-pungency cultivars was significantly higher compared to the control and it was decreased in the highly pungent cultivars under drought stress. Ruiz-Lau et al. [1] determined that in highly pungent chilli peppers, capsaicin synthase activity was reduced in response to water stress, and this effect depended on both stress severity and fruit age. The decrease in capsaicin synthase activity was probably the reason for the significant reduction of capsaicinoids in Jolokia fruits collected from plants that had undergone drought stress compared to control and fruits subjected to 34 days of stress in comparison with those stressed for 24 days. The remaining cultivars did not show the significant changes in capsaicinoid content over a two-week interval.

The cultivars of chilli pepper examined in our research responded to drought stress in different ways. The fruit pungency of ‘Bird´s Eye’ and ‘Puerto Rican’ slightly increased under 21-day-long drought stress application, and then later decreased. ‘Aji Lemon Drop’ showed the opposite behaviour and ‘Jolokia’ had increased fruit pungency under drought stress regardless of its duration. Similar behaviour was described by González-Zamora et al. [7] for temperature stress. This observation confirms common mechanisms of pepper reaction to different abiotic stresses. The fact that only ‘Jolokia’ showed significant changes in capsaicin content could be connected with fruit size. Since ‘Jolokia’ is a medium-fruited cultivar we can agree with Gurung et al. [28], who concluded that small-fruited cultivars are less affected by environment compared to the medium- and large-fruited ones. Temperature and water availability are not the only factors influencing capsaicinoids’ accumulation in pepper fruits, as fruit size also has an effect. According to Othman et al. [29], capsaicin content in chilli peppers depended on the age of the fruit and the light conditions. Generally, the increase in SHU values under drought stress in the present experiment was lower compared to other trials, with temperature stress reported by González-Zamora et al. [7] and Phimchan et al. [19]. 

Chilli pepper fruits are an excellent source of AsA, one of the most effective antioxidants, although the genetic factor is the most important determinant of the final amount of this compound. In the present study, the lowest amount of ascorbic acid was detected in ‘Aji Lemon Drop’ and the highest in ‘Jolokia’ fruits. Kopta et al. [30] stated that ‘Aji Lemon Drop’ reached the amount of around 600 mg AsA per 1000 g^−1^ of fruit fresh weight, which is consistent with our results. ‘Jolokia’ was mentioned in the research of Kantar et al. [25], who reported the AsA amount of 383 mg 1000 g^−1^ of FW, and this range is considerably narrower than our findings. AsA content in ‘Bird´s Eye’ fruits varied between 530 and 1590 mg 1000 g^−1^ FW [31] and is therefore in line with the results of our research.

Generally, drought increased AsA content in chilli pepper fruits, and this effect was more significant after 21-day-long stress application. According to Tuteja et al. [32], it could be expected that drought stress would trigger the increased biosynthesis of major antioxidant compounds such as AsA. It has also been reported that stress-tolerant genotypes contain higher endogenous levels of AsA [33]. However, Munné-Bosch and Alegre [34] have stated that drought stress caused increased AsA content in chloroplasts but a general reduction of this compound in the leaf tissues of several plant species. Bartoli et al. [35] did not find a correlation between drought stress and AsA biosynthesis, although Seminario et al. [36] showed that water deficit caused a reduction in the biosynthesis of AsA in soybean. The present research showed that there were differences in the stress duration needed to reduce the content of AsA, which could possibly point to cultivars with improved tolerance to drought. Based on this relation, from our research, ‘Bird´s Eye’ can be pointed to as a drought-tolerant cultivar. This cultivar did not show a decrease in AsA content even after longer stress application.

### 3.2. Stress Parameters of Chilli Pepper Fruits and Leaves

Malondialdehyde (MDA) is the final product of lipid peroxidation in the cellular membranes. The increased MDA level reflects oxidative membrane damage [12]. The activities of antioxidant enzymes, and the DPPH˙ scavenging activity increased under drought in chilli pepper leaves and fruits, although the intensity of the reaction varied among the cultivars used in the present research. ‘Jolokia’, the highly pungent cultivar, with fruits rich in AsA, showed the highest antioxidant activity related to capsaicinoids and AA compounds. Fruits of ‘Aji Lemon Drop’, characterised by the lowest SHU values and lowest antioxidant enzyme activities, showed about 50% lower DPPH˙ scavenging activity, although total phenols content was highest in the fruits of this cultivar. The hierarchical cluster analysis showed the greatest distance between ‘Jolokia’ and ‘Aji Lemon Drop’, taking into account both leaf and fruit trials. It can be concluded that capsaicinoids and AsA are responsible for antioxidant activity of highly pungent peppers such as ‘Jolokia’ under the conditions of our experiment, although the importance of phenolic compounds in the contribution to the total antioxidant activity of peppers, suggested by Bogusz et al. [37], could be significant in mildly pungent genotypes such as ‘Aji Lemon Drop’. Asnin and Park [38] stated that the capsaicin profile did not directly reflect antioxidant activity, due to the presence of other antioxidant compounds, i.e., phenolics. Moreover, phenolic compounds, because of their antioxidant activity, can play a certain role in the prevention of civilisational diseases. The phenolic content in chilli pepper cultivars investigated by Bogusz et al. [37] was between 1078 and 4992 µg GAE (gallic acid equivalents) g^−1^ FW, and this range corresponds with the results of our research and confirms that the investigated cultivars are valuable sources of phenolics in the human diet. Drought often causes oxidative stress in plants, leading to an increase in the amounts of polyphenols and other secondary metabolites responsible for the acclimation response [38,39]. In the study of Zhuang et al. [8], significant correlations were shown between total phenolic contents and antioxidant activity in all nine pepper cultivars.

Soluble carbohydrates (SC) play a crucial role, not only in metabolic pathways, and they are important quality parameters of pepper fruits [40]. It is interesting that significant differences in SC were determined only for fruits of the ‘Aji Lemon Drop’ and ‘Jolokia’ cultivars, which reacted in opposite ways to drought conditions. ‘Aji Lemon Drop’ showed a decrease in SC level in drought-stressed fruits, while ‘Jolokia’ had an increase. SC could be involved in the drought tolerance mechanisms of ‘Jolokia’ as osmoprotectants. The roles of soluble carbohydrates have been widely accepted as osmoprotectants against drought and other environmental stresses, regulating osmotic adjustment, providing membrane protection and scavenging reactive oxygen species [40].

Drought stress first causes a morphological plant response, namely the closure of stomata in order to minimise transpiration. This causes a decrease of intercellular carbon dioxide concentration due to ongoing photosynthesis in the light. The decreased availability of CO_2_ stimulates ribulose-1,5-bisphosphate oxygenation and thus photorespiratory hydrogen peroxide (H_2_O_2_) production in the peroxisomes [41]. Enzymatic components involved in antioxidant defence comprise of catalase (CAT), ascorbate peroxidase (APX) and guaiacol peroxidase (GPX) which scavenge H_2_O_2_ into oxygen and water [42]. Antioxidant enzyme activities in chilli pepper fruits and leaves were higher under drought stress conditions. In most cases, differences were statistically significant but the remaining cases showed a similar tendency. Drought stress application involved the enzymatic mechanisms of free radical scavenging in all the investigated cultivars, acting in both leaves and fruits but with different intensities in particular cultivars. ‘Aji Lemon Drop’ fruits needed the most intensive enzymatic protection because oxidative membrane damage (MDA) was highest in the fruits of this cultivar. In general, all the analysed enzymes contributed equally to the protective mechanisms against reactive oxygen species in chilli pepper fruits, which was reflected in the principal component analysis (PCA) graph showing closely clustering eigenvectors of these trials. According to Laxa et al. [41], APX, CAT and GPX represent the principal reactive oxygen species scavengers in plants. APX and CAT are activated more quickly and strongly in tolerant genotypes compared to sensitive ones. In contrast, sensitive genotypes activate GPX more intensively than tolerant ones. Based on the cited reference, the investigated chilli pepper cultivars developed effective enzymatic mechanisms to scavenge reactive oxygen species under stress conditions.

## 4. Materials and Methods 

### 4.1. Experimental Design

The experiment took place in 2019 in a polycarbonate greenhouse of the Faculty of Horticulture, Mendel University in Brno, Czech Republic, in the municipality of Lednice (GPS 48.79 N, 16.80 E). The experimental site is located at 176 m above sea level. Meteorological parameters during the vegetation period (June–October) were as follows: the average temperature was 1.6 °C, average relative humidity was 68.9% and the period of sunlight was 1196 h [43].

Structural parameters of the polycarbonate greenhouse were as follows: length 15, width 6 m, height 2.5 m, ultraviolet- stabilised polycarbonate, thickness 5 mm. 

The experimental protocol was based on the factorial combination between four cultivars and two irrigation regimes, using a split plot design with six replicates. 

The chilli pepper cultivars used for the experiment were *Capsicum chinense* Jacq.´Jolokia´ and ´Puerto Rican´, *C. annuum* L.→´Bird´s Eye´ and *C. baccatum* L. ´Aji Lemon Drop´. All cultivars were provided by the company World of Chilli, Czech Republic. Seedlings were planted on 10 June in 10 litre pots with the substrate Agro Profimix 2 (Agro C.S., Česká Skalice, Czech Republic). 

The two levels of the irrigation regime corresponded to: (i) the control, 35–60% of volumetric water content (VWC), and (ii) drought stress, 18–28% VWC. Drought conditions were set on the basis of soil daily VWC measurement with ECH2O-TE (Decagon Device, Brno-Černovice, Czech Republic). VWC setting corresponds to the appropriate level of growing media humidity to assure optimal plant growth. According to the actual VWC, the plants were separately irrigated. Drought conditions were applied for 34 days (2 September–7 October). Measurements of analytical parameters were carried out at 13 and 21 days after drought stress application, respectively. The total amount of water applied to the control plants was 10 L per plant, on average. Drought-treated plants received 60% less water.

Pests and diseases were monitored and treated according to the standard recommendation for the pepper crops. Prior to transplanting, the fertilisation was practiced by supplying to each plant 68 kg N, 20 P, 75 K, 58 Ca. During the cultivation, fertilisation was regularly applied in a form of foliar spraying with 1g L^−1^ of Kristalon (YARA Agri, Staré Město, Czech Republic), containing N:P:K 20:5:10, and Mg 2%.

Fruits were harvested from uniform plants at the stage of physiological maturity. Analytical parameters (ascorbic acid and SHU) were evaluated from the first (24 September) and second (7 October) harvests. Stress metabolites (total phenolics, soluble carbohydrates, DPPH˙ radical scavenging activity, malondialdehyde and antioxidant enzymes) were analysed separately in leaves and fruits sampled on 7 October. Analyses were performed in the Department of Horticulture, University of Agriculture in Krakow, Poland.

### 4.2. Ascorbic Acid (AsA) and Scoville Heat Unit (SHU) Evaluation

Only fruit pulp was analysed. The high-performance liquid chromatography (HPLC) method according to Sawant et al. [44] was used for the determination of ascorbic acid. The analyses were performed by Reversed phaseHPLC in a C18 column at 254 nm using a UV-vis detector (ECOM, České Meziříčí, Czech Republic). The results were calculated in the fresh weight (FW). For HPLC analysis of capsaicin in fruits, the official AOAC Method 995.03 (Association of Official Agricultural Chemists) was used [45] with the above-mentioned HPLC system at 280 nm wavelength. In order to calculate Scoville heat units (SHU), the capsaicin and dihydrocapsaicin content (ug g^−1^) was multiplied by 15. 

### 4.3. Total Phenolics

Total phenolics (TP) were estimated with the modified Folin–Ciocalteu colorimetric method [46]. Fresh leaf and fruit samples of 2.0 g were mixed with 10 cm^3^ of 80% methanol and centrifuged (3492 g, 10 min). The supernatant (0.1 cm^3^) was mixed with 2 cm^3^ of sodium carbonate. Then, after 2 min, 0.1 cm^3^ Folin–Ciocalteu’s reagent mixed with deionised water (1:1 v/v) was added to the test tubes. The absorbance of the resulting blue colour was measured at 750 nm using a UV-VIS (Ultraviolet–visible spectroscopy) Helios Beta spectrophotometer (Waltham, MA, USA) against a reference solution. The results were expressed as gallic acid equivalents (GAE), mg GAE per 1 g FW.

### 4.4. Soluble Carbohydrates

Total soluble carbohydrates were determined with the Yemm and Willis [47] method. Leaf and fruit samples were mixed with 80% ethanol and anthrone reagent, then the absorbance was measured at 625 nm with a UV-VIS Helios Beta spectrophotometer.

### 4.5. DPPH˙ Radical Scavenging Activity

DPPH˙ radical scavenging activity was determined according to the Bartosz [48] using the artificial 2,2-diphenyl-1-picrylhydrazyl radical (DPPH˙). A mixture of fresh leaf and fruit samples (2.0 g) were ground and centrifuged (3492 g, 10 min) with 80% methanol. Test tubes contained 0.1 cm^3^ of supernatant and 4.9 cm^3^ of 0.1 mM DPPH˙ dissolved in 80% methanol. The mixture was incubated for 15 min in the dark and at 20 °C, the absorbance was measured using the UV-VIS Helios Beta spectrophotometer at 517 nm. DPPH˙ radical scavenging activity was calculated as DPPH˙ (%) = ((A0 − A1)/A0) × 100, where A0 and A1 are the absorbances of the reference and test solutions, respectively.

### 4.6. Malondialdehyde

The malondialdehyde (MDA) content was measured by the thiobarbituric acid method [49]. Fresh leaf and fruit samples were homogenised in 0.1% trichloroacetic acid (TCA) and centrifuged at 13,968 g for 15 min. The reaction mixture consisted of the supernatant, 0.1 M K-phosphate buffer of pH 7.6, and 0.5% thiobarbituric acid dissolved in 20% TCA. The absorbance of the mixture was measured at 532 nm and corrected for non-specific turbidity by subtracting the absorbance at 600 nm (UV-VIS Helios Beta spectrophotometer). The amount of MDA was calculated from the difference in absorbance at these wavelengths, using a molar absorbance coefficient of 155 mM^−1^ cm^−1^.

### 4.7. Antioxidant Enzyme Assays

Catalase (CAT, EC 1.11.1.6) activity was determined with the method of Aebi [50]. Samples of 2.5 g fresh leaves and fruits were ground in an ice bath with 20 cm^3^ 0.05 M potassium phosphate buffer and centrifuged (3492 g, 15 min, 4 °C). Supernatant (0.2 cm^3^) was added to test tubes containing 1.8 cm^3^ 0.05 M phosphate buffer (pH 7.0) and 1.0 cm^3^ 0.05% H_2_O_2_ solution in 0.05 M potassium phosphate buffer (pH 7.0). The measurement of H_2_O_2_ disappearance was made spectrophotometrically at 240 nm (UV-VIS Helios Beta spectrophotometer).

Ascorbate peroxidase (APX, EC 1.11.1.11) and guaiacol peroxidase (GPX, EC 1.11.1.7) were determined with the methods of Nakano and Asada [51] and Zhang et al. [52], respectively. 2 g fresh weight leaf samples were homogenised in 50 mM potassium phosphate buffer (pH 7.0) containing 1 mM ethylene diamine tetraacetic acid (EDTA), 1% soluble polyvinyl pyrrolidone, and 1 mM phenylmethylsulfonyl fluoride in ice bath at 4 °C. The mixture was centrifuged at 13,968 g for 15 min. The reaction mixture for APX determination contained 50 mM potassium phosphate buffer (pH 7.0), 0.5 mM ascorbate, 0.1 mM H_2_O_2_, and 0.15 cm^3^ of supernatant. The H_2_O_2_-dependent oxidation of ascorbate was measured as the decrease in absorbance at 290 nm (ε = 2.8 mM^−1^ cm^−1^) with a UV-VIS Helios Beta spectrophotometer. APX activity was expressed as µg AsA min^−1^ g^−1^ FM. The reaction mixture for GPX determination contained 50 mM phosphorus buffer (pH 7.0), H_2_O_2_ (1%), and guaiacol (4%) and 0.2 cm^3^ of supernatant. The increase in absorbance at 470 nm due to guaiacol oxidation was recorded at 25 °C using a UV-VIS Helios Beta spectrophotometer over a period of 3 min (ε = 26.6 mM^−1^ cm^−1^). GPX activity was presented as µmol tetraguaiacol min^−1^ g^−1^ FM.

### 4.8. Statistical Data Evaluation

Data were statistically processed by analysis of variance (ANOVA) at a significance level of *p ≤* 0.05. If significant differences were detected between the treatments, the averages were compared and the mean separation was performed using the Scheffe’s test through the Statistica 12.0 software package (StatSoft Inc., Tulsa, USA). The correlation coefficient *r* was calculated to determine the relation between biochemical traits. Hierarchical cluster analysis was performed on the standardised data using a measure of Euclidean distance and the Ward minimum variance method. Experimental data were also processed using principal component analysis (PCA), in order to evaluate the existing relationships with original variables.

## 5. Conclusions

In the present experiment, the evaluation of chilli pepper cultivars in terms of nutritional features and drought tolerance have given interesting hints for commercial use or breeding. It can be stated that SHU and AsA content were significantly affected by all the three experimental factors (cultivar, drought stress and drought duration). The application of moderate drought stress to pot-grown chilli pepper activated biochemical and physiological adaptation mechanisms in cultivar-dependent ways. The drought-induced changes in metabolic pathways were different in the leaves and fruits of investigated genotypes. In some cultivars, short-term drought stress led to an increase in pungency and ascorbic acid content, whereas longer lasting drought resulted in the reverse effect. In ‘Jolokia’, the differences in pungency and ascorbic acid were statistically significant for both drought durations. Moderate stress levels could be an interesting tool for modifying the chemical profile in selected chilli cultivars. Interestingly, drought stress elicited the activation of the antioxidant system which represents an important goal, along with the identification of appropriate metabolic pathways, i.e., specific enzymes synthesis, connected with plant tolerance to drought. Indeed, the expressing of specific enzymes can lead to a beneficial increase in drought tolerance. The analysis of stress-induced metabolites in chilli pepper cultivars, namely capsaicinoids, ascorbic acid and total phenols, as well as antioxidant enzyme activities, can provide detailed information on common or specific mechanisms of drought tolerance and allow us to develop promising genotypes for broad practical application.

## Figures and Tables

**Table 1:** Results from analysis of variance (ANOVA) relevant to Scoville Heat Units (SHU) and ascorbic acid content as affected by cultivar, drought stress and drought duration.

ANOVA Source of Variation	SHU	Ascorbic Acid
Cultivar (C)	***	***
Drought stress (DS)	***	***
Drought duration (DD)	***	***
C × DS	***	***
C × DD	***	***
DS × DD	ns	***
C × DS × DD	***	***

*** *p* ≤ 0.001; ns, not significant. The mean separation was performed using Scheffe’s test.

**Table 2:** The total phenols, soluble carbohydrates and DPPH˙ (2,2-diphenyl-1-picrylhydrazyl) scavenging activity in chilli pepper leaves and fruits as affected by cultivar and drought stress application.

Interaction		Total Phenols (mg GAE g^−1^ FW)		Soluble Carbohydrates (g 100 g^−1^ FW)		DPPH (% Scavenging of DPPH˙)	
Cultivar	Irrigation Regime	Fruits	Leaves	Fruits	Leaves	Fruits	Leaves
Aji Lemon Drop	Control	1.81c ^1^	0.60f	14.63b	3.77b	43.4e	34.9d
	Drought	1.81c	0.82e	12.75c	4.04ab	39.3e	36.6d
Jolokia	Control	3.03a	1.19c	14.09b	5.31ab	71.9b	90.3a
	Drought	3.18a	1.39a	15.84a	5.98a	90.8a	89.8a
Bird’s Eye	Control	2.65b	0.69f	8.50d	3.36b	64.8c	34.9d
	Drought	2.71b	0.98d	9.25d	4.94ab	72.6b	50.2c
Puerto Rican	Control	2.47b	1.30ab	6.28e	4.57ab	52.2d	78.9b
	Drought	2.52b	1.18c	8.33e	5.21ab	84.6a	87.7a
**Main Effects**							
Irrigation regime: Control Drought		2.49 2.55	0.91B 1.09A	11.29 11.54	4.22 5.00	58.62B 71.83A	58.0B 66.1A
		ns		ns	ns		
Cultivar: Aji Lemon Drop Jolokia Bird’s Eye Puerto Rican		1.81D 3.11A 2.68B 2.49C	0.71D 1.29A 0.83C 1.23B	7.51D 14.96A 8.87C 13.70B	3.90ns 5.65ns 4.15ns 4.96ns	41.4C 81.35A 68.7B 68.7B	35.8D 90.0A 42.5C 84.2B

^1^ Means of three replicates; data were subjected to two-way ANOVA; means within a column followed by different letters (capital letters for main effects and lowercase letters for interactions) are significantly different at *p* ≤ 0.05 according to Scheffe’s test; ns, not significant differences.

**Table 3:** Catalase, ascorbate peroxidase, guaiacol peroxidase and malondialdehyde in chilli pepper leaves and fruits as affected by cultivar and drought stress application.

Interaction		Catalase (μmol H_2_O_2_ min^−1^ g^−1^ FW)		Ascorbate Peroxidase (µg AsA min^−1^ g^−1^ FW)		Guaiacol Peroxidase (μmol of tetraguaiacol min^−1^ g^−1^ FW)		Malondialdehyde (μmol g^−1^ FW)	
Cultivar	Irrigation Regime	Fruits	Leaves	Fruits	Leaves	Fruits	Leaves	Fruits	Leaves
Aji Lemon Drop	Control	6.006b ^1^	7.19d	0.173b	0.251c	0.472a	1.285c	51.7b	53.9e
	Drought	9.294a	9.95c	0.221a	0.361a	0.526a	1.739b	61.4a	87.3b
Jolokia	Control	0.558c	8.17d	0.127c	0.202d	0.337b	0.998c	41.9de	53.2e
	Drought	0.641c	10.30b	0.146c	0.267c	0.472a	1.179c	44.7c	68.7c
Bird’s Eye	Control	0.850c	6.84d	0.030e	0.077f	0.129d	3.165b	38.9e	50.5e
	Drought	1.971c	9.40c	0.168b	0.184e	0.233c	1.200c	43.2d	92.3a
Puerto Rican	Control	0.644c	10.03b	0.030e	0.201d	0.117d	1.216c	43.4d	39.5f
	Drought	2.228c	12.11a	0.079d	0.294b	0.270c	3.165a	46.9c	62.1d
**Main Effects**									
Irrigation regime: Control Drought		2.014B 3.533A	8.06B 10.4A	0.090B 0.153A	0.183B 0.277A	0.264B 0.375A	1.170B 1.824A	44.0B 49.1A	49.3B 77.6A
Cultivar: Aji Lemon Drop Jolokia Bird’s Eye Puerto Rican		7.650A 0.599B 1.411B 1.436B	8.57B 9.24B 8.12C 11.07A	0.197A 0.137B 0.099C 0.054D	0.306A 0.235B 0.131C 0.248B	0.499A 0.404B 0.193C 0.181C	1.512B 1.089C 2.182A 1.205C	56.6A 43.3C 41.1D 45.2B	70.58A 60.96B 71.45A 50.81C

^1^ Means of three replicates; data were subjected to two-way ANOVA; means within a column followed by different letters (capital letters for main effects and lowercase letters for interactions ) are significantly different at *p ≤* 0.05 according to Scheffe’s test.

**Table 4:** Pearson’s correlation coefficients (r) between all studied parameters of pepper fruits (N = 24).

	SHU	AsA	TP	SC	DPPH	APX	GPX	CAT	MDA
SHU	1.000								
AsA	0.706 ***^,1^	1.000							
TP	0.800 ***	0.886 ***	1.000						
SC	0.550 **	−0.153	0.037	1.000					
DPPH	0.634 ***	0.750 ***	0.842 ***	0.036	1.000				
APX	0.022	−0.530 **	−0.350	0.678 ***	−0.248	1.000			
GPX	0.213	−0.444 *	−0.317	0.864 ***	−0.170	0.841 ***	1.000		
CAT	−0.531 **	−0.894 ***	−0.854 ***	0.293	−0.701 ***	0.694 ***	0.654 **	1.000	
MDA	−0.386	−0.748 ***	−0.763 ***	0.339	−0.588 **	0.706 ***	0.941 ***	0.283	1.000

^1^,***, ** and *—significant at *p* ≤ 0.001, 0.01 and 0.05 levels, respectively. SHU—Scoville Heat Units; AsA—ascorbic acid; TP—total phenols; SC—soluble carbohydrates; DPPH—% scavenging of DPPH˙; APX—ascorbate peroxidase; GPX—guaiacol peroxidase; CAT—catalase; MDA—malondialdehyde.

**Table 5:** Pearson’s correlation coefficients (r) between all studied parameters of pepper leaves (N = 24).

	TP	SC	DPPH	APX	GPX	CAT	MDA
TP	1.000						
SC	0.793 ***^,1^	1.000					
DPPH	0.933 ***	0.774 ***	1.000				
APX	0.167	0.236	0.138	1.000			
GPX	−0.567 **	−0.615 **	−0.594 **	−0.503 *	1.000		
CAT	0.636 **	0.555 **	0.563 **	0.556 **	−0.476 *	1.000	
MDA	−0.123	0.105	−0.277	0.412 **	−0.124	0.283	1.000

^1^,***, ** and *—significant at *p* ≤ 0.001, 0.01 and 0.05 levels, respectively. TP—total phenols; SC—soluble carbohydrates; DPPH—% scavenging of DPPH˙; APX—ascorbate peroxidase; GPX—guaiacol peroxidase; CAT—catalase; MDA—malondialdehyde.
